# Analysis of the success rate of conversion using ibutilide administration in radiofrequency catheter ablation of persistent atrial fibrillation and its effects on postoperative recurrence

**DOI:** 10.1186/s12872-024-03787-1

**Published:** 2024-02-20

**Authors:** Meijuan Li, Xiping Liu, Yan Zhang, Weibin Huang, Bingbo Hou, Sen Huang, Feng Gao

**Affiliations:** 1 Department of Cardiovascular Medicine, Zhongshan Hospital Xiamen University, Xiamen, 361000 Fujian Province China; 2 Department of Cardiovascular Medicine, The First Hospital of Fuzhou, Fuzhou, 350000 Fujian Province China

**Keywords:** Ibutilide, Persistent atrial fibrillation, Catheter ablation, Recurrence, Influencing factors

## Abstract

**Objective:**

To assess the efficacy of ibutilide administration during radiofrequency catheter ablation of persistent atrial fibrillation (AF), to explore the success rate of conversion and related influential factors, and to analyze the effects of ibutilide on postoperative recurrence.

**Methods:**

A total of 192 patients with persistent AF who underwent catheter ablation from January 1, 2019, to December 31, 2021. These patients failed in conversion of AF to normal sinus rhythm by intraoperative catheter ablation. Patients were categorized into effective group (115 cases) and ineffective group (77 cases) based on whether sinus rhythm was restored after application of ibutilide.

**Results:**

The overall success rate of conversion using ibutilide administration was 59.9%. The success rate was associated with weight ((68.12 ± 11.72 vs. 72.83 ± 12.08) kg, *P* = 0.008), the duration of AF ((34.67 ± 55.68 vs. 66.52 ± 95.21) months, *p* = 0.008), diameter of left atrium (LAD) ((44.39 ± 5.80 vs. 47.36 ± 6.10) mm,*P* = 0.002), and N-terminal pro-brain natriuretic peptide (NT-proBNP) level ((854.85 ± 770.84 vs. 662.88 ± 659.18) pg/ml,*P* = 0.030). The results showed the duration of AF was associated with early recurrence, while early recurrence was not a risk factor for late recurrence. And duration of AF was associated with postoperative maintenance time of normal sinus rhythm, whereas successful conversion into normal sinus rhythm using ibutilide administration had no influence on postoperative maintenance time of normal sinus rhythm.

**Conclusion:**

Ibutilide showed to be effective in catheter ablation of AF, the success rate of conversion was correlated with the duration of AF, LA diameter, and NT-proBNP level. Besides, the duration of AF was found as a risk factor for early postoperative recurrence, while ibutilide administration for successful conversion had no influence on predicting postoperative recurrence and had no influence on postoperative maintenance time of sinus rhythm.

**Supplementary Information:**

The online version contains supplementary material available at 10.1186/s12872-024-03787-1.

## Introduction

Atrial fibrillation (AF) is one of the most common arrhythmias, and it has gradually attracted clinicians’ attention. Radiofrequency, cryoballoon catheter ablation, and antiarrhythmic drug therapy are therapeutic options for AF. Ablation is effective at reducing recurrent atrial arrhythmias and also in the reduction of AF burden. However, after radiofrequency catheter ablation of AF, a significant proportion of patients have still AF, thus, intraoperative conversion of sinus rhythm is required, and the conversion rate of the traditional class III antiarrhythmic drug (amiodarone) is low [1], while class III antiarrhythmic drug (ibutilide) is an effective drug, rapidly converting AF to normal sinus rhythm [2]. The present study aimed to assess the effectiveness of ibutilide administration during radiofrequency catheter ablation for persistent AF, to explore the success rate of conversion and related influential factors, and to analyze the effects of ibutilide on postoperative recurrence.

## Methods

### Study subjects

Consecutive patients who underwent radiofrequency catheter ablation for persistent atrial fibrillation and failed to convert to sinus rhythm intraoperatively (regardless of previous radiofrequency ablation) were enrolled from January 1, 2019, to December 31, 2021, in a total of 192 cases, of which 141 were male,51 were female. All patients had regularly received anticoagulation for at least 3 weeks before surgery, and no left atrial thrombus was detected by esophageal ultrasonography. All patients stopped taking antiarrhythmic drugs for more than 5 half-lives. The exclusion criteria were as follows: (i) contraindication to anticoagulation or the left atrial thrombus; (ii) diameter of left atrium (LA) > 55 mm; (iii) hyperthyroidism; (iv) unstable angina, acute myocardial infarction; (v) the feasibility of converting AF to normal sinus rhythm; (vi) chest X-ray indicating acute pulmonary congestion, heart failure, or the New York Heart Association (NYHA) grade ≥ III.

### Research flow diagram (Fig. 1)

**Fig. 1:**
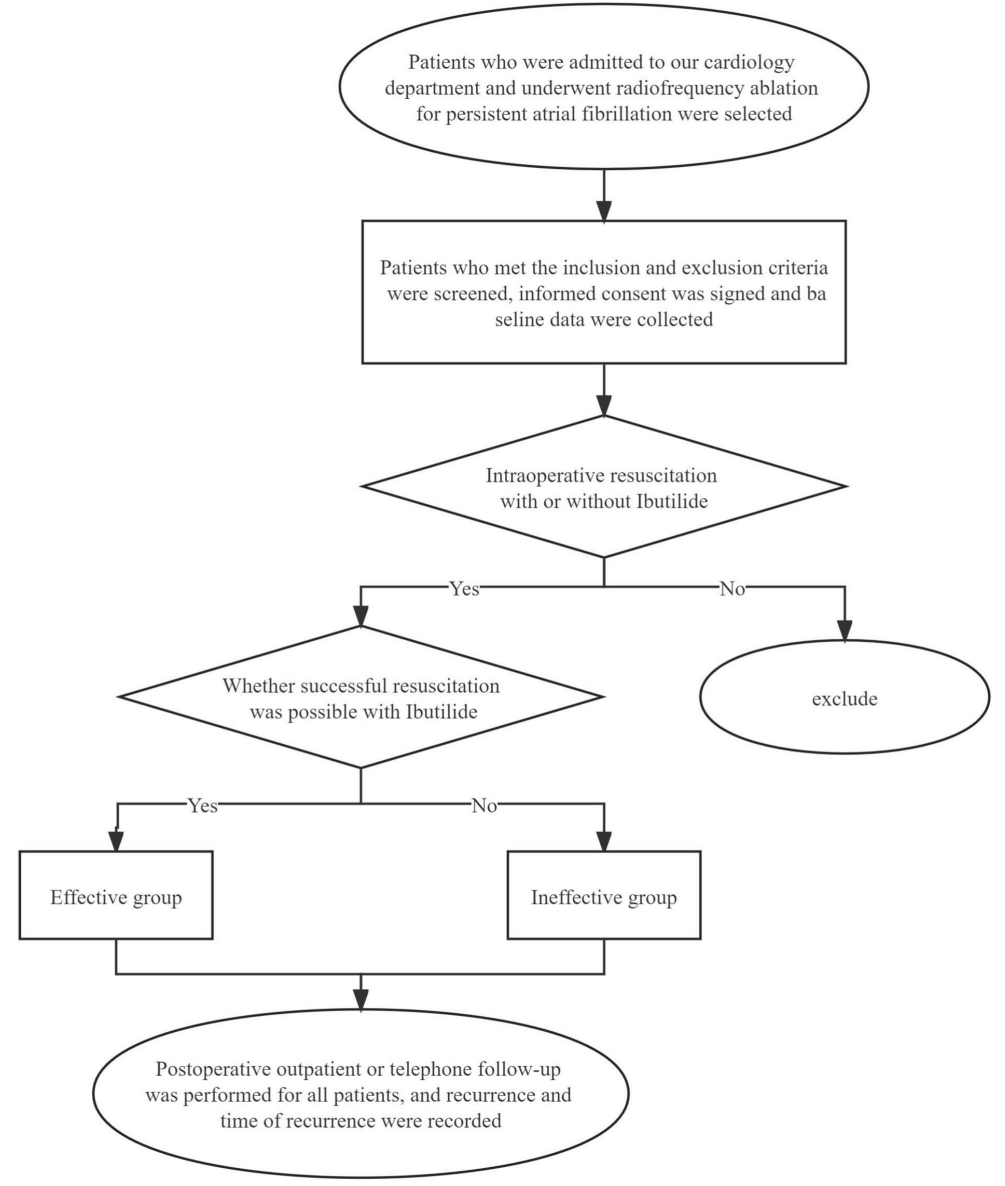
Basic flow diagram of the study

### Ablation therapy

Bilateral annular pulmonary vein ablation was performed in all patients under the guidance of Carto or Ensite 3D calibration system,until the bilateral pulmonary vein potentials disappeared. and in our center, for persistent or long-standing persistent atrial fibrillation, we continued to perform linear ablation of the left atrial apex, the mitral isthmus endocardial and/or epicardial surfaces, and the tricuspid isthmus, that is, 2C3L, and if atrial fibrillation could not be terminated, we continued to perform fragmentation potential ( CAFE) ablation, and superior vena cava isolation. If normal sinus rhythm could not be restored after the above-mentioned ablation protocol, ibutilide (1 mg for body weight ≥ 60 kg or 0.01 mg/kg for body weight < 60 kg) was given intraoperatively and diluted with 10 ml of physiological saline for 10 min, and observed for 30 min. If normal sinus rhythm could not be recovered, electrical cardioversion (EC) was given, and ablation was performed for patients with atrial tachycardia and atrial flutter until sinus rhythm was recovered. If EC or radiofrequency catheter ablation of atrial tachycardia or atrial flutter was required, the procedure was considered as unsuccessful or invalid.

### Verification of restoration of normal sinus rhythm

After radiofrequency ablation or after the application of Ibutilide, the ablation endpoint was verified when atrial fibrillation was reverted to sinus rhythm. The pulmonary vein potential was measured by the Lasso electrode, combined with coronary sinus electrode pacing, to verify whether a bidirectional conduction block between the pulmonary vein and the left atrium was achieved; to verify whether the mitral isthmus line, tricuspid isthmus line, and left atrial apex line showed bidirectional block, and to end the procedure if the ablation endpoint was achieved.

Follow-up method: All patients were followed up by outpatient department or telephone, including: whether there were chest tightness, palpitations, dizziness, chest pain and other suspicious symptoms indicating atrial fibrillation or similar symptoms with preoperative atrial fibrillation, 12-lead ECG and 24-hour dynamic ECG examination results, whether regular medication was used, and whether medication plans were adjusted.

All patients were required to have a 12-lead ECG at the nearest medical institution immediately when suspicious symptoms indicating atrial fibrillation occurred. Even if there is no relevant symptom attack, routine ECG examination and 24-hour dynamic ECG examination and monitoring are also required 1, 3 and 6 months after ablation and every 6 months thereafter. If the patient has a recurrence of atrial fibrillation, record the time from the end of ablation to recurrence.

### Statistical analysis

In the present study, SPSS 24.0 software (IBM, Armonk, NY, USA) was used to perform the statistical analysis, the measurement data were expressed as mean ± standard deviation (x ± s), and the count data were expressed as number of cases and percentage. The Kolmogorov-Smirnov test was performed for the assessment of normal distribution of measurement data, and the independent-samples t-test was used for the analysis of normally distributed measurement data. For abnormally distributed data, the Mann-Whitney U test was used. For count data, the Pearson’s Chi-square test, Yates’ continuity correction of the Chi-square test, the Fisher’s exact test were used for making comparison between two groups, which according to the total sample size and the size of the theoretical minimum sampling frequency. The Cox proportional-hazards regression was employed to analyze variables that were significantly associated with AF-free survival after ablation therapy, and the hazard ratio (HR) was calculated. Kaplan-Meier curves and log-rank tests were applied to assess the statistical differences between the effective ibutilide and ineffective ibutilide groups. *P* < 0.05 was considered statistically significant.

## Results

### Patients’ general data

A total of 192 patients (male (141) vs. female (51)) who underwent radiofrequency catheter ablation for persistent AF from January 1, 2019, to December 31, 2021, and failed in conversion into normal sinus rhythm intraoperatively were included. According to the effectiveness of intraoperative administration of ibutilide, patients were divided into effective ibutilide (*n* = 115) and ineffective ibutilide (*n* = 77) groups, with an overall conversion success rate of 59.9%. There was no significant difference in age, duration of AF, gender, heart failure, hypertension, diabetes, coronary artery disease, stroke history, peripheral vascular disease, and CHA_2_DS_2_-VASc score between the two groups (*P* > 0.05). The success rate of conversion using ibutilide was related to weight ((68.12 ± 11.72 vs. 72.83 ± 12.08) kg, *P* = 0.008),the duration of AF ((34.67 ± 55.68 vs. 66.52 ± 95.21) months, *P* = 0.008), the diameter of LA ((44.39 ± 5.80 vs. 47.36 ± 6.10) mm,*P* = 0.002), and N-terminal pro-brain natriuretic peptide (NT-proBNP) level ((854.85 ± 770.84 vs. 662.88 ± 659.18) pg/ml,*P* = 0.030). The differences were all statistically significant, as shown in Table 1.

**Table 1:** Comparison of patients’ demographic and clinical characteristics between the two groups

	Effective ibutilide group(*n* = 115)	Ineffective ibutilide group(*n* = 77)	*P*
Age (years old)	64.46 ± 11.02	61.26 ± 11.72	0.852
Weight(kg)	68.12 ± 11.72	72.83 ± 12.08	0.008*
Duration of atrial fibrillation (months)	34.67 ± 55.68	66.52 ± 95.21	0.008*
Male	80(69.6%)	61(79.2%)	0.138
Female	35(30.4%)	16(20.8%)	
Heart failure	11(9.6%)	10(13.0%)	0.457
Hypertension	40(34.8%)	37(48.0%)	0.066
Diabetes	24(21.1%)	12(15.6%)	0.358
Coronary heart disease	13(11.3%)	11(14.3%)	0.540
Stroke history	9(7.8%)	9(11.7%)	0.368
Peripheral vascular disease	27(23.5%)	19(24.7%)	0.849
CHA_2_DS_2_-VAScscore	1.96 ± 1.70	2.16 ± 1.71	0.377
LA diameter(mm)	44.39 ± 5.80	47.36 ± 6.10	0.002*
EF(%)	60.48 ± 8.30	61.19 ± 9.23	0.384
NT-proBNP baseline level(pg/mL)	854.85 ± 770.84	662.88 ± 659.18	0.030*

*P > 0.05 indicates that the difference is not statistically significant, and *P* < 0.05 indicates that the difference is statistically significant

### Analysis of the effectiveness of ibutilide administration in radiofrequency catheter ablation

All patients enrolled in the study underwent bilateral radiofrequency catheter ablation of the circumflex pulmonary vein and 2C3L for persistent or long-standing persistent AF (192 patients), with CAFE-guided ablation and SVC isolation if necessary. Afterwards, AF was successfully converted to normal sinus rhythm by intraoperative administration of ibutilide (1 mg for body weight ≥ 60 kg or 0.01 mg/kg for body weight < 60 kg), with a total success rate of 59.9%. One case was still in persistent AF after the DC resuscitation (2–8 times), which led to the unsuccessful operation, and for the other patients, conversion to normal sinus rhythm was successfully conducted after electrical resuscitation.

### Analysis of early recurrence and late recurrence after administration of ibutilide

In the present study, AF recurrence was defined as a rapid atrial arrhythmia of ≥ 30 s confirmed by electrocardiogram (ECG) or ECG after ablation. The onset of AF and atrial flutter within 3 months after radiofrequency catheter ablation was defined as early recurrence, and the onset of AF and atrial flutter after 3 months was defined as late recurrence. All patients were postoperatively followed up, postoperative recurrence and duration of recurrence were recorded, and the risk factors affecting postoperative recurrence were analyzed.

All patients were followed up in outpatient clinics or by telephone after surgery, and there were no lost cases during the follow-up process, with a median follow-up time of 14 months, including a median follow-up time of 15 months in the ibutilide-effective group and 12 months in the ibutilide-ineffective group, which were not statistically different from each other (*P* = 0.249).

#### Univariable analysis of postoperative early recurrence

The univariable analysis of postoperative early recurrence suggested that the duration of AF was a risk factor for postoperative early recurrence, Statistically significant difference in duration of atrial fibrillation between early recurrence and non-recurrence groups (86.24 ± 119.42 vs. 42.17 ± 66.20, *P* < 0.05), while the success rate of conversion using ibutilide and other factors did not predict early recurrence of AF (Table 2).

**Table 2:** Univariable analysis of postoperative early recurrence

	Early recurrence (*n* = 23)	Early non-recurrence(*n* = 169)	*P*
Age (years old)	62.74 ± 10.94	61.20 ± 11.34	0.457
Weight(kg)	70.13 ± 8.83	69.98 ± 12.45	0.954
Duration of atrial fibrillation (months)	86.24 ± 119.42	42.17 ± 66.20	0.020*
Male	15(65.2%)	126(74.6%)	0.341
Female	8(34.8%)	43(25.4%)	
Heart failure	3(13.0%)	18(10.7%)	1.000
Hypertension	10(43.5%)	67(39.6%)	0.725
Diabetes	4(17.4%)	32(18.9%)	1.000
Coronary heart disease	4(17.4%)	20(11.8%)	0.674
Stroke history	3(13.0%)	15(8.9%)	0.793
Peripheral vascular disease	6(26.1%)	40(23.7%)	0.799
CHA_2_DS_2_-VASc score	2.35 ± 2.08	1.99 ± 1.65	0.555
LA diameter(mm)	46.54 ± 6.64	45.43 ± 6.02	0.469
EF(%)	60.91 ± 9.53	60.74 ± 8.57	0.790
NT-proBNP(pg/mL)	783.53 ± 689.68	779.00 ± 741.13	0.907
Success rate of conversion using ibutilide	10(43.5%)	105(62.1%)	0.087
Failure rate of conversion using ibutilide	13(56.5%)	64(37.9%)	

*P > 0.05 indicates that the difference is not statistically significant, and *P* < 0.05 indicates that the difference is statistically significant

Because only 1 risk factor was found in the univariable analysis and the *P* value of the remaining factors was greater than 0.20, which was not suitable for inclusion in the multivariate analysis, no further multivariate analyses were performed in this study.

#### Univariable analysis of postoperative late recurrence

The univariable analysis of postoperative late recurrence suggested that no statistical association between early recurrence and late recurrence (i.e., early recurrence did not predict late recurrence). Similarly, the success rate of conversion using ibutilide and other factors did not predict late recurrence of AF, as shown in Table 3.

**Table 3:** Univariable analysis of late recurrence

	Late recurrence (*n* = 33)	Late non-recurrence (*n* = 159)	*P*
Age (years old)	59.82 ± 12.88	61.70 ± 10.93	0.471
Weight(kg)	73.18 ± 12.47	69.33 ± 11.90	0.095
Duration of atrial fibrillation (months)	42.66 ± 49.39	48.44 ± 79.96	0.537
Male	25(75.8%)	116(73.0%)	0.740
Female	8(24.2%)	43(27.0%)	
Heart failure	2(6.1%)	19(11.9%)	0.497
Hypertension	12(36.4%)	65(40.9%)	0.630
Diabetes	4(12.1%)	32(20.1%)	0.284
Coronary heart disease	3(9.1%)	21(13.2%)	0.718
Stroke history	5(15.2%)	13(8.2%)	0.356
Peripheral vascular disease	7(21.2%)	39(24.5%)	0.685
CHA_2_DS_2_-VASc score	1.88 ± 1.75	2.07 ± 1.70	0.468
LA diameter(mm)	45.56 ± 5.35	45.56 ± 6.24	0.794
EF(%)	61.66 ± 6.32	60.58 ± 9.08	0.881
NT-proBNP(pg/mL)	696.32 ± 602.12	796.08 ± 757.23	0.575
Early relapse	1(3.0%)	22(13.8%)	0.148
Success rate of conversion using ibutilide	21(63.6%)	94(59.1%)	0.630
Failure rate of conversion using ibutilide	12(36.4%)	65(40.9%)	

*P > 0.05 indicates that the difference is not statistically significant, and *P* < 0.05 indicates that the difference is statistically significant

### Analysis of factors influencing early recurrence in the effective ibutilide and ineffective ibutilide groups

The results showed that only the duration of AF was a risk factor for early recurrence in patients in the ineffective ibutilide group (91.92 ± 100.43 vs. 61.37 ± 94.10, *P* < 0.05), while the same conclusion was not observed in the effective ibutilide group (Table 4).

**Table 4:** Analysis of factors influencing early recurrence in the ineffective ibutilide group

	Early recurrence(*n* = 13)	Early non-recurrence(*n* = 64)	*P*
Age (years old)	65.31 ± 10.07	60.44 ± 11.93	0.149
Weight(kg)	69.77 ± 9.36	73.46 ± 12.54	0.319
Duration of atrial fibrillation (months)	91.92 ± 100.43	61.37 ± 94.10	0.048*
Male	9(69.2%)	52(81.2%)	0.549
Female	4(30.8%)	12(18.8%)	
Heart failure	3(23.1%)	7(10.9%)	0.463
Hypertension	7(53.8%)	30(46.9%)	0.646
Diabetes	3(23.1%)	9(14.1%)	0.691
Coronary heart disease	3(23.1%)	8(12.5%)	0.576
Stroke history	2(15.4%)	7(10.9%)	1.000
Peripheral vascular disease	4(30.8%)	15(23.4%)	0.837
CHA_2_DS_2_-VASc score	2.85 ± 1.77	2.01 ± 1.67	0.091
LA diameter(mm)	48.17 ± 5.86	47.21 ± 6.19	0.587
EF(%)	61.62 ± 9.90	61.10 ± 9.17	0.872
NT-proBNP(pg/mL)	683.08 ± 474.12	658.78 ± 694.04	0.550

*P > 0.05 indicates that the difference is not statistically significant, and *P* < 0.05 indicates that the difference is statistically significant

### Analysis of postoperative maintenance time of normal sinus rhythm in AF patients

**Fig. 2:**
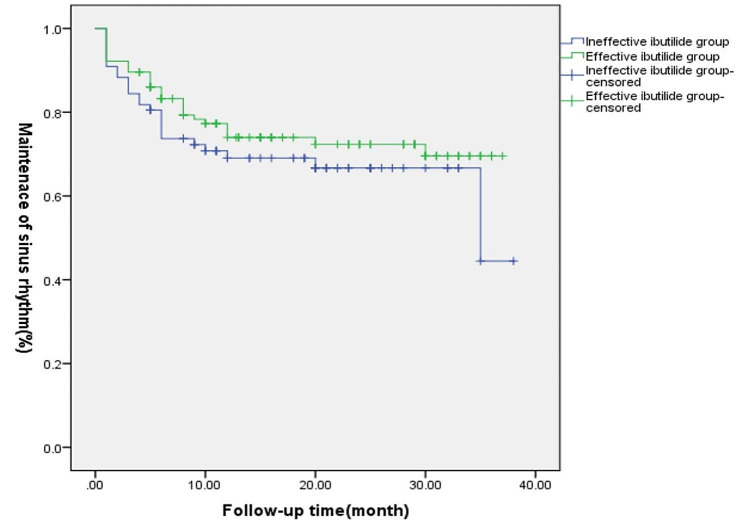
Effect of the application of ibutilide in the effective and ineffective groups on the maintenance of sinus rhythm in patients. Ineffective ibutilide group (*n* = 77),Effective ibutilide group (*n* = 115),*P* = 0.291. *P* > 0.05, indicating that the difference is not statistically significant

The COX proportional-hazards regression was applied to analyze factors influencing the postoperative maintenance time of normal sinus rhythm in AF patients. For count data ,equiproportionate risk was judged by quadratic log survival plot in COX regression, and factors that did not meet the assumption of equiproportionate risk (peripheral vascular disease) were excluded. For measurement data, ROC curves were first plotted to determine whether there was a correlation between the variables and recurrence, and the Yordon index was calculated to find the optimal cutoff point. Ultimately, only AF duration (AUC = 0.612, *P* = 0.016) was included in the statistics, with a cutoff value of AF duration ≥ 15 months. An equiproportional risk judgment was performed suggesting compliance with the equiproportional risk assumption, and a COX test was performed, with the final result suggesting that AF duration ≥ 15 months was a risk factor for AF recurrence (*p* = 0.003),as shown in Table 5, while the effectiveness of intraoperative ibutilide administration, other factors had no effect on the time to maintain normal sinus rhythm after surgery (*P* > 0.05). Further analysis of the effectiveness of intraoperative ibutilide administration using Kaplan-Meier curves and log-rank tests also yielded the same conclusion (*P* = 0.291) (Fig. 2).

**Table 5:** Factors influencing patients’ survival time

	Risk ratio(HR)	95%CI	*P*
Ibutilide	0.756	0.444–1.287	0.303
Duration of atrial fibrillation ≥ 15 months	2.426	1.355–4.343	0.003*
Gender	1.181	0.659–2.115	0.577
Heart failure	0.926	0.369–2.238	0.871
Hypertension	1.000	0.583–1.716	1.000
Diabetes	0.692	0.327–1.465	0.336
Coronary heart disease	1.039	0.470–2.296	0.925
Stroke history	1.870	0.882–3.966	0.103

*P > 0.05 indicates that the difference is not statistically significant, and *P* < 0.05 indicates that the difference is statistically significant

## Discussion

Among clinical arrhythmias, atrial fibrillation is extremely common, and a variety of factors: such as advanced age, coronary heart disease, hypertension, diabetes, cardiomyopathy, and obesity can lead to atrial electrical remodeling and structural remodeling, which triggers atrial fibrillation [3]. With the advancement of catheter ablation technology for atrial fibrillation and the improvement of safety, more and more patients choose radiofrequency catheter ablation for atrial fibrillation. Pulmonary vein isolation is the most common method for radiofrequency ablation of atrial fibrillation, and pulmonary vein potential isolation is a good endpoint for ablation. For persistent atrial fibrillation or long-range persistent atrial fibrillation, 2C3L combined with fractionated potential ablation and superior vena cava isolation is mostly used. However, a considerable number of patients still have AF after linear isolation during radiofrequency catheter ablation and can’t restore sinus rhythm, so sinus rhythm needs to be actively converted during surgery and pharmacological cardioversion or electrical cardioversion can be used.

Although catheter ablation alone can inhibit atrial fibrillation, it sometimes can not be converted to sinus rhythm during surgery, resulting in prolonged conversion time and decreased successful conversion rate. Pharmacologic cardioversion is a safe and effective method to convert sinus rhythm and control symptoms. Ibutilide is a class III antiarrhythmic agent that prolongs the action potential duration [4] in the atrial myocardium. Therefore, it is commonly used for the conversion of atrial fibrillation and other arrhythmias. The electrophysiological mechanisms of cardioversion are mainly as follows: ① By inhibiting potassium channels, it specifically inhibits potassium currents, but the difference is that ibutilide also promotes sodium and calcium influx in the plateau phase and plays a role in prolonging the plateau phase of action potential in cardiomyocytes; it causes prolongation of action potential duration and prolongation of QTc in cardiomyocytes, and then exerts its antiarrhythmic effect [5, 6]. ② Ibutilide is more effective in inhibiting atrial myocardium than sinus node, atrioventricular node and ventricular myocardium, so it is more effective in inhibiting atrial arrhythmia than ventricular arrhythmia [7]. Especially in new-onset atrial fibrillation, ibutilide is effective and has low direct side effects [8], and it has been shown that intraoperative application of low-dose ibutilide avoids or reduces additional matrix ablation after circumferential pulmonary vein isolation in patients with persistent atrial fibrillation without compromising long-term clinical success [9]. Intraoperative application of ibutilide can prolong atrial muscle action potential duration and strengthen the inhibition of atrial myocardium, thereby inhibiting atrial fibrillation, improving successful conversion rate, and reducing postoperative recurrence rate. In addition, the application of ibutilide can shorten the ablation time to a certain extent and reduce the damage of myocardial cells.

Compared with amiodarone, ibutilide is more effective in cardioversion of atrial fibrillation,increasing the success rate of electrical conversion and reducing the energy of electrical conversion, improving the success rate of surgery [10]. Cardioversion time was significantly shorter than amiodarone, the incidence of cardiovascular adverse reactions was significantly higher with ibutilide than with amiodarone [11]. In the literature, ibutilide has been reported to have a conversion rate of 30 to 50% for atrial fibrillation and up to 70 to 90% for atrial flutter [12]. Our study found that the success rate of conversion after a single dose of ibutilide was 59.9% in patients who failed to convert sinus rhythm during radiofrequency ablation of atrial fibrillation, which was higher than the success rate reported in the literature,it may be related to the intraoperative application of 2C3L procedure, and the success rate of conversion could be improved after the administration of ibutilide.

At present, many domestic and foreign scholars have studied the rational use of ibutilide in atrial fibrillation ablation. Aktas M K et al. [13] published a study involving 89 patients and concluded that the use of ibutilide in AF ablation was safe and effective. Chinese scholars have found that the success rate of immediate conversion after intraoperative application of ibutilide is about 60%, the success rate of conversion after a single dose of ibutilide after catheter ablation of persistent atrial fibrillation is 48.8%, and the total power of conversion after repeated administration is about 72.1% [14, 15]. However, there are still few studies on the intraoperative application of ibutilide efficacy to predict postoperative recurrence of atrial fibrillation.Therefore, this study focuses on the relationship between the success rate of ibutilide conversion and postoperative recurrence. In this study, we found that the overall success rate of conversion of sinus rhythm with ibutilide during radiofrequency ablation of atrial fibrillation was 59.9%. The success rate of ibutilide conversion was related to patient’s weight, the duration of atrial fibrillation, LA, and NT-proBNP levels. As for AF recurrence, early recurrence was defined as episodes of AF and atrial flutter within 3 months after radiofrequency ablation; late recurrence was defined as episodes of AF and atrial flutter after 3 months [16].Our study suggests that: Duration of AF is a risk factor for early recurrence after ablation, but early recurrence is not a risk factor for late recurrence. The results of survival analysis showed that the duration of atrial fibrillation was associated with the time to maintain sinus rhythm after ablation, but whether ibutilide was successfully converted during ablation did not affect the time to maintain sinus rhythm after ablation.

In the conclusion of this study, intraoperative application of ibutilide was not beneficial for postoperative recurrence of atrial fibrillation, which may be related to the fact that the effect of ibutilide was temporary and did not affect the long-term maintenance mechanism of atrial fibrillation. In fact, similar studies by other scholars have reached similar conclusions. Sun et al. [9]studied the effect of intraoperative combined use of ibutilide on the ablation and clinical outcomes after atrial fibrillation ablation in 135 patients with persistent atrial fibrillation, and the results showed that whether or not to use ibutilide in the ablation of persistent atrial fibrillation had no difference on the postoperative recurrence rate of patients.SM Singh et al. [17] published a study on the application of ibutilide in guiding CAFÉ ablation of persistent atrial fibrillation, which included a total of 200 patients with atrial fibrillation who were randomized to receive ibutilide or placebo before CAFÉ potential ablation. After a 12-month follow-up, it was found that there was no difference in the recurrence rate of postoperative atrial fibrillation between the two groups.Although the success rate of ibutilide conversion did not predict AF ablation outcomes. However, these findings do not represent that ibutilide has no role in predicting AF ablation outcomes. He [18]et al performed sequential low-dose (0.004 mg/kg) intravenous injection of ibutilide in 180 patients with persistent atrial fibrillation and further PVI and atrial matrix modification if necessary, and the results showed that patients with persistent atrial fibrillation who could terminate persistent atrial fibrillation by pulmonary vein isolation alone ± low-dose ibutilide during ablation had a significantly better incidence of postoperative arrhythmia freedom than patients with persistent atrial fibrillation who required further matrix modification, which indicates a good prognosis. Preoperative sequential low-dose ibutilide testing is a simple way to screen this group of patients. Therefore, we can predict recurrence after ablation based on the patient ‘s response to preoperative intravenous ibutilide injection and help the operator make decisions about ablation strategy.

This study still has some limitations. First, this study is a single-center study, and its results need to be repeated in a multicenter trial. and the sample size of this study is small, with a median follow-up time of 14 months, which is relatively insufficient, and its follow-up time should also be appropriately prolonged; second, this study did not investigate the conversion of ibutilide dose to sinus rhythm after catheter ablation for persistent atrial fibrillation or long-range persistent atrial fibrillation, and whether high dose or low dose has a better conversion effect needs to be further explored.In addition, if the occurrence of early recurrence is observed in the study, the use of antiarrhythmic medications will be adjusted, and the adjustment of medications may have an impact on the occurrence of late recurrence, which is also a limitation of this study.

The results of this study may predict intraoperative response to ibutilide conversion by patient’s weight, the duration of atrial fibrillation, LA, and NT-proBNP levels, and duration of atrial fibrillation can predict early postoperative recurrence, helping to stratify the risk of atrial fibrillation recurrence and individualize the management of patients with atrial fibrillation. Patients with persistent atrial fibrillation who have left atrial scars and areas of low voltage occupying a large proportion of the left atrial area have a higher recurrence rate [19, 20] and often require left atrial matrix modification to achieve good ablation results. However, for patients with persistent atrial fibrillation, matrix ablation, which is widely used, not only increases the operation time and radiation exposure rate, but also increases the risk of atrial tachycardia.For patients with persistent atrial fibrillation receiving PVI and linear ablation combined with ibutilide when necessary, if the conversion to sinus rhythm is not successful, it indicates that there may be a complex matrix, and it is often necessary to combine other ablation methods to improve the ablation success rate. Therefore, the preoperative and intraoperative combined use of ibutilide can, to a certain extent, distinguish the severity of atrial remodeling in patients with persistent atrial fibrillation, thus guiding operators to choose appropriate ablation strategies to achieve better ablation outcomes.

## Conclusion

Ibutilide showed to be effective in catheter ablation of AF, the success rate of conversion was correlated with the weight, duration of AF, LA diameter, and NT-proBNP level. Besides, the duration of AF was found as a risk factor for early postoperative recurrence, while ibutilide administration for successful conversion had no influence on predicting postoperative recurrence and had no influence on postoperative maintenance time of sinus rhythm.

### Electronic supplementary material

Below is the link to the electronic supplementary material.

Supplementary Material 1

## Data Availability

All data generated or analysed during this study are included in this published article [and its supplementary information files].
