# Supplementary material for: Analysis of the success rate of conversion using ibutilide administration in radiofrequency catheter ablation of persistent atrial fibrillation and its effects on postoperative recurrence
Source: BMC Cardiovasc Disord. 2024 Feb 20;24:118. doi: 10.1186/s12872-024-03787-1 (PMC10877843; doi:10.1186/s12872-024-03787-1)
Supplement: Supplementary file 1 — Supplementary Material 1 [file 12872_2024_3787_MOESM1_ESM.pdf]

Supplementary Table 1:Raw data for analysis

Number	Date	Surgery time	Sex（Female＝1，Male＝0）	Age	Duration of atrial fibrillation（Month）	Heart failure	Hypertension	Diabetes	Coronary artery disease	History of Stroke	Peripheral vascular diseases	CHA2DS2VASC评分	Diameter of left atrium	EF	2C3L	Ibutilide	osas	UA	NTproBNP baseline level	Early recurrence	Late recurrence	Recurrence	Recurrence time（Month）
1.00	667.00	2020.7.10	0.00	65.00	96.00	0.00	1.00	0.00	0.00	0.00	1.00	3.00	73.00	58.00	+box	0.00	#NULL!	455.00	550.00	0.00	0.00	0.00	#NULL!
2.00	317.00	2021.6.25	0.00	60.00	48.00	1.00	0.00	0.00	0.00	0.00	0.00	1.00	59.00	47.00	+svc	0.00	#NULL!	399.00	232.00	1.00	0.00	1.00	3.00
3.00	986.00	2019.8.26	0.00	81.00	24.00	0.00	0.00	0.00	0.00	0.00	0.00	2.00	58.00	41.00	1	0.00	0.00	376.00	4210.00	0.00	0.00	0.00	#NULL!
4.00	746.00	2020.4.22	0.00	47.00	120.00	0.00	0.00	0.00	0.00	0.00	0.00	0.00	58.00	50.00	1	1.00	#NULL!	281.00	416.00	1.00	0.00	1.00	1.00
5.00	335.00	2021.6.7	0.00	50.00	84.00	0.00	0.00	0.00	0.00	0.00	0.00	0.00	57.00	60.00	1	0.00	#NULL!	450.00	217.00	0.00	0.00	0.00	#NULL!
6.00	746.00	2020.4.22	0.00	53.00	96.00	0.00	0.00	1.00	1.00	0.00	1.00	3.00	57.00	39.00	1	1.00	#NULL!	327.00	1410.00	0.00	0.00	0.00	20.00
7.00	634.00	2020.8.12	0.00	54.00	1.00	0.00	1.00	0.00	0.00	0.00	0.00	1.00	56.00	67.00	+box	0.00	#NULL!	511.00	#NULL!	0.00	0.00	0.00	#NULL!
8.00	1068.00	2019.6.5	0.00	62.00	6.00	0.00	1.00	0.00	0.00	0.00	0.00	1.00	56.00	64.00	1	1.00	0.00	496.00	955.00	0.00	0.00	0.00	#NULL!
9.00	128.00	2021.12.31	1.00	60.00	12.00	1.00	0.00	0.00	0.00	0.00	0.00	2.00	56.00	50.00	1	1.00	#NULL!	439.00	#NULL!	0.00	0.00	0.00	#NULL!
10.00	1142.00	2019.3.23	0.00	56.00	120.00	0.00	0.00	0.00	0.00	0.00	1.00	2.00	55.00	69.00	1	0.00	0.00	430.00	217.00	0.00	1.00	1.00	6.00
11.00	898.00	2019.11.22	0.00	72.00	120.00	0.00	0.00	0.00	1.00	0.00	1.00	3.00	55.00	68.00	1	0.00	#NULL!	454.00	836.00	0.00	0.00	0.00	#NULL!
12.00	657.00	2020.7.20	1.00	73.00	120.00	0.00	1.00	1.00	1.00	0.00	1.00	6.00	55.00	57.00	1	0.00	#NULL!	626.00	517.00	1.00	0.00	1.00	2.00
13.00	653.00	2020.7.24	0.00	62.00	1.00	0.00	0.00	0.00	0.00	0.00	0.00	0.00	55.00	63.00	1	0.00	#NULL!	500.00	662.00	0.00	0.00	0.00	#NULL!
14.00	382.00	2021.4.21	0.00	76.00	60.00	0.00	1.00	1.00	0.00	0.00	0.00	4.00	55.00	61.00	1	0.00	#NULL!	330.00	437.00	0.00	0.00	0.00	#NULL!
15.00	461.00	2021.2.1	1.00	71.00	60.00	0.00	0.00	0.00	0.00	0.00	1.00	3.00	55.00	50.00	+café	1.00	#NULL!	604.00	1230.00	0.00	0.00	0.00	#NULL!
16.00	195.00	2021.10.25	0.00	76.00	1.00	0.00	0.00	0.00	0.00	0.00	0.00	2.00	55.00	58.00	1	1.00	#NULL!	327.00	1061.00	0.00	0.00	0.00	#NULL!
17.00	1005.00	2019.8.7	1.00	68.00	6.00	0.00	1.00	0.00	1.00	0.00	1.00	5.00	54.00	64.00	1	1.00	0.00	489.00	1770.00	0.00	0.00	0.00	#NULL!
18.00	928.00	2019.10.23	0.00	69.00	0.30	0.00	0.00	0.00	0.00	0.00	0.00	1.00	54.00	64.00	1	1.00	0.00	393.00	717.00	0.00	0.00	0.00	#NULL!
19.00	690.00	2020.6.17	0.00	53.00	84.00	0.00	1.00	0.00	0.00	0.00	0.00	1.00	53.00	62.00	1	0.00	#NULL!	488.70	858.00	0.00	0.00	0.00	#NULL!
20.00	643.00	2020.8.3	0.00	56.00	60.00	0.00	1.00	1.00	0.00	1.00	0.00	4.00	53.00	52.00	1	0.00	#NULL!	369.00	1488.00	0.00	1.00	1.00	10.00
21.00	461.00	2021.2.1	0.00	66.00	48.00	0.00	1.00	0.00	0.00	0.00	1.00	3.00	53.00	50.00	1	0.00	1.00	576.00	1049.00	0.00	1.00	1.00	4.00
22.00	690.00	2020.6.17	0.00	51.00	0.30	0.00	1.00	0.00	0.00	0.00	0.00	1.00	53.00	37.00	1	1.00	#NULL!	440.00	2977.00	0.00	0.00	0.00	#NULL!
23.00	494.00	2020.12.30	0.00	65.00	0.13	0.00	1.00	0.00	0.00	0.00	0.00	2.00	53.00	62.00	1	1.00	#NULL!	424.00	280.00	0.00	0.00	0.00	#NULL!
24.00	723.00	2020.5.15	0.00	66.00	1.00	0.00	0.00	0.00	0.00	0.00	0.00	1.00	52.00	66.00	+Marshall	0.00	#NULL!	264.00	1654.00	0.00	0.00	0.00	#NULL!
25.00	389.00	2021.4.14	0.00	72.00	24.00	1.00	0.00	0.00	0.00	0.00	1.00	3.00	52.00	75.00	1	0.00	#NULL!	240.00	186.00	1.00	0.00	1.00	1.00
26.00	376.00	2021.4.27	1.00	67.00	48.00	0.00	0.00	0.00	0.00	1.00	0.00	4.00	52.00	60.00	1	0.00	#NULL!	340.00	1776.00	1.00	0.00	1.00	1.00
27.00	1142.00	2019.3.23	0.00	63.00	36.00	0.00	1.00	0.00	0.00	0.00	1.00	2.00	52.00	55.00	1	1.00	0.00	425.00	251.00	0.00	1.00	1.00	8.00
28.00	1007.00	2019.8.5	1.00	55.00	84.00	0.00	0.00	0.00	0.00	1.00	0.00	3.00	52.00	64.00	1	1.00	0.00	386.00	566.00	0.00	1.00	1.00	3.00
29.00	622.00	2020.8.24	0.00	60.00	24.00	0.00	1.00	0.00	0.00	0.00	0.00	1.00	51.00	62.00	1	0.00	#NULL!	459.00	926.00	1.00	0.00	1.00	3.00
30.00	365.00	2021.5.8	0.00	68.00	480.00	0.00	1.00	0.00	0.00	0.00	0.00	2.00	51.00	67.00	1	0.00	#NULL!	456.00	393.00	0.00	0.00	0.00	#NULL!
31.00	174.00	2021.11.15	0.00	68.00	192.00	1.00	1.00	0.00	0.00	0.00	0.00	3.00	51.00	65.00	+box	0.00	#NULL!	381.00	439.00	0.00	0.00	0.00	#NULL!
32.00	912.00	2019.11.8	0.00	71.00	10.00	0.00	1.00	1.00	0.00	0.00	0.00	3.00	51.00	57.00	1	1.00	#NULL!	427.00	710.00	0.00	0.00	0.00	#NULL!
33.00	604.00	2020.9.11	1.00	68.00	60.00	0.00	1.00	1.00	1.00	1.00	1.00	8.00	51.00	65.00	+svc	1.00	#NULL!	291.00	1223.00	1.00	0.00	1.00	1.00
34.00	319.00	2021.6.23	0.00	64.00	60.00	0.00	0.00	0.00	0.00	0.00	0.00	0.00	51.00	71.00	1	1.00	#NULL!	472.00	249.00	1.00	0.00	1.00	1.00
35.00	220.00	2021.9.30	0.00	55.00	60.00	1.00	0.00	0.00	0.00	0.00	0.00	1.00	51.00	58.00	1	1.00	#NULL!	561.00	#NULL!	0.00	1.00	1.00	5.00
36.00	604.00	2020.9.11	0.00	50.00	24.00	0.00	0.00	0.00	0.00	0.00	0.00	0.00	50.00	65.00	1	0.00	#NULL!	377.00	133.00	0.00	0.00	0.00	#NULL!
37.00	602.00	2020.9.13	0.00	76.00	60.00	0.00	1.00	0.00	1.00	0.00	1.00	5.00	50.00	72.00	1	0.00	#NULL!	205.00	996.00	0.00	0.00	0.00	#NULL!
38.00	599.00	2020.9.16	1.00	68.00	3.00	1.00	0.00	0.00	0.00	1.00	1.00	6.00	50.00	38.00	1	0.00	#NULL!	321.00	#NULL!	0.00	0.00	0.00	#NULL!
39.00	291.00	2021.7.21	0.00	67.00	36.00	0.00	0.00	0.00	0.00	0.00	0.00	1.00	50.00	62.00	1	0.00	#NULL!	449.00	540.00	0.00	0.00	0.00	#NULL!
40.00	172.00	2021.11.17	0.00	38.00	3.00	0.00	0.00	1.00	0.00	0.00	1.00	2.00	50.00	73.00	1	0.00	#NULL!	555.00	2895.00	0.00	0.00	0.00	#NULL!
41.00	991.00	2019.8.21	1.00	61.00	12.00	0.00	1.00	0.00	0.00	0.00	0.00	2.00	50.00	43.00	1	1.00	0.00	378.00	1564.00	0.00	0.00	0.00	#NULL!
42.00	921.00	2019.10.30	0.00	54.00	120.00	0.00	0.00	0.00	0.00	0.00	0.00	0.00	50.00	57.00	1	1.00	#NULL!	670.00	508.00	0.00	0.00	0.00	#NULL!
43.00	706.00	2020.6.1	1.00	69.00	48.00	0.00	1.00	1.00	1.00	0.00	1.00	6.00	50.00	69.00	1	1.00	#NULL!	423.00	2994.00	0.00	1.00	1.00	5.00
44.00	590.00	2020.9.25	0.00	59.00	24.00	0.00	0.00	0.00	0.00	0.00	0.00	0.00	50.00	66.00	1	1.00	#NULL!	382.00	361.00	0.00	1.00	1.00	6.00
45.00	354.00	2021.5.19	1.00	71.00	2.00	1.00	0.00	0.00	0.00	0.00	1.00	4.00	50.00	68.00	+café	1.00	#NULL!	417.00	4761.00	0.00	0.00	0.00	#NULL!
46.00	170.00	2021.11.19	0.00	53.00	0.50	1.00	1.00	0.00	0.00	0.00	0.00	2.00	50.00	79.00	1	1.00	#NULL!	275.00	751.00	0.00	0.00	0.00	#NULL!
47.00	1049.00	2019.6.24	1.00	64.00	24.00	0.00	0.00	0.00	0.00	0.00	0.00	1.00	49.00	64.00	+box&svc&café	0.00	0.00	413.00	223.00	0.00	1.00	1.00	6.00
48.00	513.00	2020.12.11	0.00	63.00	24.00	0.00	0.00	0.00	0.00	0.00	0.00	0.00	49.00	55.00	1	0.00	#NULL!	395.00	115.00	0.00	1.00	1.00	6.00
49.00	349.00	2021.5.24	0.00	72.00	12.00	0.00	1.00	0.00	1.00	0.00	1.00	4.00	49.00	70.00	1	0.00	#NULL!	427.00	1214.00	0.00	0.00	0.00	#NULL!

50.00	251.00	2021.8.30	0.00	82.00	360.00	0.00	1.00	1.00	1.00	1.00	1.00	8.00	49.00	55.00	1	0.00	#NULL!	#NULL!	1032.00	0.00	0.00	0.00	#NULL!
51.00	900.00	2019.11.20	1.00	65.00	84.00	0.00	1.00	0.00	0.00	0.00	0.00	3.00	49.00	59.00	1	1.00	#NULL!	327.00	681.00	0.00	1.00	1.00	6.00
52.00	870.00	2019.12.20	1.00	67.00	84.00	0.00	1.00	1.00	0.00	1.00	0.00	6.00	49.00	65.00	+svc	1.00	#NULL!	306.00	1141.00	0.00	0.00	0.00	#NULL!
53.00	676.00	2020.7.1	0.00	64.00	24.00	1.00	0.00	0.00	0.00	0.00	0.00	1.00	49.00	45.00	1	1.00	#NULL!	416.00	377.00	0.00	0.00	0.00	#NULL!
54.00	536.00	2020.11.18	1.00	83.00	24.00	1.00	0.00	0.00	0.00	0.00	0.00	4.00	49.00	64.00	1	1.00	#NULL!	586.00	1677.00	0.00	0.00	0.00	#NULL!
55.00	473.00	2021.1.20	0.00	72.00	12.00	1.00	0.00	0.00	1.00	0.00	1.00	4.00	49.00	49.00	1	1.00	#NULL!	478.00	607.00	0.00	0.00	0.00	#NULL!
56.00	433.00	2021.3.1	1.00	78.00	60.00	0.00	1.00	0.00	1.00	0.00	1.00	6.00	49.00	64.00	2C	1.00	#NULL!	305.00	1185.00	0.00	1.00	1.00	6.00
57.00	781.00	2020.3.18	0.00	57.00	2.00	0.00	1.00	0.00	0.00	0.00	0.00	1.00	48.00	46.00	1	0.00	#NULL!	713.00	713.00	0.00	0.00	0.00	#NULL!
58.00	599.00	2020.9.16	1.00	77.00	7.00	0.00	1.00	1.00	0.00	0.00	0.00	5.00	48.00	69.00	1	0.00	#NULL!	346.00	354.00	1.00	0.00	1.00	1.00
59.00	482.00	2021.1.11	1.00	70.00	36.00	0.00	1.00	0.00	0.00	0.00	0.00	3.00	48.00	60.00	+box	0.00	#NULL!	250.00	1383.00	0.00	0.00	0.00	#NULL!
60.00	471.00	2021.1.22	0.00	52.00	120.00	0.00	1.00	0.00	0.00	0.00	0.00	1.00	48.00	58.00	1	0.00	#NULL!	445.00	174.00	0.00	0.00	0.00	#NULL!
61.00	431.00	2021.3.3	0.00	44.00	2.00	0.00	1.00	0.00	0.00	0.00	0.00	1.00	48.00	67.00	1	0.00	#NULL!	457.00	80.20	0.00	0.00	0.00	#NULL!
62.00	342.00	2021.5.31	1.00	67.00	12.00	0.00	1.00	0.00	0.00	0.00	0.00	3.00	48.00	59.00	1	0.00	#NULL!	283.00	#NULL!	0.00	0.00	0.00	#NULL!
63.00	321.00	2021.6.21	0.00	63.00	60.00	0.00	1.00	0.00	0.00	0.00	0.00	1.00	48.00	69.00	1	0.00	#NULL!	474.00	120.00	1.00	0.00	1.00	3.00
64.00	1089.00	2019.5.15	1.00	64.00	24.00	0.00	0.00	0.00	0.00	0.00	0.00	1.00	48.00	39.00	+svc	1.00	0.00	348.00	1607.00	1.00	0.00	1.00	1.00
65.00	1010.00	2019.8.2	0.00	72.00	36.00	0.00	1.00	0.00	0.00	1.00	0.00	4.00	48.00	64.00	1	1.00	0.00	508.00	725.00	0.00	0.00	0.00	#NULL!
66.00	748.00	2020.4.20	0.00	66.00	36.00	0.00	1.00	1.00	0.00	0.00	0.00	3.00	48.00	64.00	1	1.00	#NULL!	465.00	1165.00	0.00	0.00	0.00	#NULL!
67.00	489.00	2021.1.4	0.00	84.00	36.00	0.00	1.00	0.00	0.00	0.00	0.00	3.00	48.00	45.00	1	1.00	#NULL!	474.00	1426.00	0.00	1.00	1.00	10.00
68.00	466.00	2021.1.27	0.00	56.00	12.00	0.00	1.00	0.00	0.00	0.00	0.00	1.00	48.00	53.00	2C	1.00	#NULL!	444.00	154.00	0.00	0.00	0.00	#NULL!
69.00	390.00	2021.4.13	0.00	62.00	48.00	0.00	0.00	1.00	0.00	1.00	1.00	4.00	48.00	62.00	1	1.00	#NULL!	322.00	455.00	0.00	0.00	0.00	#NULL!
70.00	233.00	2021.9.17	0.00	38.00	0.03	0.00	0.00	0.00	0.00	0.00	0.00	0.00	48.00	42.00	1	1.00	#NULL!	629.00	419.00	0.00	0.00	0.00	#NULL!
71.00	185.00	2021.11.4	0.00	74.00	120.00	0.00	0.00	0.00	0.00	0.00	1.00	2.00	48.00	54.00	2C	1.00	#NULL!	360.00	1272.00	0.00	0.00	0.00	#NULL!
72.00	648.00	2020.7.29	0.00	68.00	36.00	0.00	1.00	0.00	0.00	1.00	0.00	4.00	47.00	65.00	1	0.00	#NULL!	412.00	227.00	0.00	1.00	1.00	20.00
73.00	550.00	2020.11.4	0.00	71.00	0.50	0.00	1.00	0.00	0.00	0.00	0.00	2.00	47.00	53.00	1	0.00	#NULL!	423.00	950.00	0.00	0.00	0.00	#NULL!
74.00	244.00	2021.9.6	0.00	63.00	9.00	0.00	1.00	0.00	0.00	0.00	0.00	1.00	47.00	57.00	2C	0.00	#NULL!	#NULL!	518.00	0.00	1.00	1.00	5.00
75.00	877.00	2019.12.13	1.00	71.00	120.00	0.00	1.00	0.00	0.00	0.00	0.00	3.00	47.00	53.00	1	1.00	#NULL!	345.00	2210.00	0.00	0.00	0.00	#NULL!
76.00	753.00	2020.4.15	0.00	58.00	12.00	0.00	0.00	1.00	0.00	0.00	0.00	1.00	47.00	69.00	1	1.00	#NULL!	568.00	170.00	0.00	1.00	1.00	12.00
77.00	508.00	2020.12.16	0.00	60.00	120.00	0.00	1.00	0.00	0.00	0.00	0.00	1.00	47.00	68.00	1	1.00	#NULL!	#NULL!	358.00	0.00	0.00	0.00	#NULL!
78.00	384.00	2021.4.19	0.00	60.00	48.00	0.00	1.00	0.00	1.00	0.00	1.00	3.00	47.00	64.00	1	1.00	#NULL!	398.00	506.00	0.00	0.00	0.00	#NULL!
79.00	240.00	2021.9.10	0.00	56.00	12.00	0.00	1.00	0.00	1.00	0.00	0.00	2.00	47.00	53.00	1	1.00	#NULL!	#NULL!	326.00	0.00	0.00	0.00	#NULL!
80.00	165.00	2021.11.24	0.00	58.00	12.00	0.00	0.00	0.00	0.00	0.00	1.00	1.00	47.00	75.00	2C	1.00	#NULL!	391.00	383.00	0.00	0.00	0.00	#NULL!
81.00	755.00	2020.4.13	0.00	64.00	12.00	0.00	1.00	0.00	1.00	0.00	1.00	3.00	46.00	59.00	1	0.00	#NULL!	379.00	190.00	0.00	0.00	0.00	#NULL!
82.00	741.00	2020.4.27	1.00	69.00	12.00	0.00	1.00	0.00	1.00	0.00	1.00	5.00	46.00	71.00	1	0.00	#NULL!	313.00	1078.00	0.00	0.00	0.00	#NULL!
83.00	590.00	2020.9.25	1.00	64.00	360.00	0.00	0.00	0.00	0.00	1.00	0.00	3.00	46.00	70.00	+box	0.00	#NULL!	271.00	611.00	0.00	0.00	0.00	#NULL!
84.00	361.00	2021.5.12	0.00	70.00	12.00	1.00	0.00	0.00	0.00	0.00	0.00	2.00	46.00	61.00	1	0.00	#NULL!	498.00	#NULL!	0.00	1.00	1.00	6.00
85.00	286.00	2021.7.26	0.00	58.00	36.00	1.00	1.00	1.00	1.00	0.00	1.00	5.00	46.00	43.00	1	0.00	#NULL!	388.00	201.00	0.00	0.00	0.00	#NULL!
86.00	1024.00	2019.7.19	0.00	61.00	2.00	0.00	1.00	1.00	0.00	0.00	0.00	2.00	46.00	63.00	1	1.00	0.00	402.00	215.00	0.00	0.00	0.00	#NULL!
87.00	874.00	2019.12.16	0.00	71.00	6.00	0.00	1.00	1.00	0.00	0.00	0.00	3.00	46.00	63.00	1	1.00	#NULL!	445.00	713.00	0.00	0.00	0.00	#NULL!
88.00	410.00	2021.3.24	0.00	78.00	48.00	0.00	1.00	1.00	0.00	0.00	0.00	4.00	46.00	63.00	2C	1.00	#NULL!	378.00	735.00	0.00	0.00	0.00	#NULL!
89.00	135.00	2021.12.24	0.00	70.00	12.00	0.00	1.00	0.00	0.00	0.00	1.00	3.00	46.00	68.00	1	1.00	#NULL!	478.00	674.00	0.00	0.00	0.00	#NULL!
90.00	550.00	2020.11.4	0.00	45.00	0.20	0.00	0.00	0.00	0.00	0.00	0.00	0.00	45.70	53.30	1	0.00	#NULL!	434.00	142.00	0.00	0.00	0.00	#NULL!
91.00	1000.00	2019.8.12	0.00	41.00	2.00	0.00	0.00	0.00	0.00	0.00	0.00	0.00	45.00	53.00	1	0.00	0.00	327.00	163.00	0.00	0.00	0.00	#NULL!
92.00	557.00	2020.10.28	0.00	55.00	6.00	1.00	0.00	0.00	0.00	0.00	0.00	1.00	45.00	39.00	1	0.00	#NULL!	401.00	1933.00	0.00	0.00	0.00	#NULL!
93.00	534.00	2020.11.20	0.00	57.00	84.00	0.00	1.00	1.00	0.00	1.00	0.00	4.00	45.00	73.00	+svc	0.00	#NULL!	537.00	843.00	1.00	0.00	1.00	1.00
94.00	317.00	2021.6.25	0.00	50.00	96.00	0.00	0.00	1.00	0.00	0.00	0.00	1.00	45.00	65.00	1	0.00	#NULL!	440.00	438.00	0.00	0.00	0.00	#NULL!
95.00	303.00	2021.7.9	0.00	72.00	0.25	0.00	0.00	0.00	0.00	0.00	0.00	1.00	45.00	72.00	1	0.00	#NULL!	328.00	871.00	0.00	0.00	0.00	#NULL!
96.00	291.00	2021.7.21	0.00	61.00	60.00	0.00	0.00	0.00	0.00	0.00	0.00	0.00	45.00	54.00	1	0.00	#NULL!	424.00	503.00	0.00	0.00	0.00	#NULL!
97.00	1133.00	2019.4.1	0.00	74.00	120.00	0.00	1.00	0.00	0.00	0.00	0.00	2.00	45.00	70.00	2C+Isthmus of TVA	1.00	0.00	477.00	1108.00	0.00	1.00	1.00	12.00
98.00	1077.00	2019.5.27	0.00	68.00	24.00	0.00	0.00	0.00	0.00	0.00	0.00	1.00	45.00	65.00	1	1.00	0.00	308.00	512.00	0.00	0.00	0.00	#NULL!
99.00	689.00	2020.6.18	0.00	66.00	6.00	0.00	0.00	0.00	0.00	0.00	0.00	1.00	45.00	75.00	1	1.00	#NULL!	460.00	1304.00	0.00	0.00	0.00	#NULL!

100.00	613.00	2020.9.2	1.00	58.00	12.00	0.00	0.00	0.00	1.00	0.00	1.00	3.00	45.00	66.00	1	1.00	#NULL!	537.00	278.00	0.00	0.00	0.00	#NULL!
101.00	535.00	2020.11.19	0.00	61.00	120.00	0.00	0.00	1.00	0.00	1.00	0.00	3.00	45.00	62.00	1	1.00	#NULL!	493.00	389.00	0.00	0.00	0.00	#NULL!
102.00	506.00	2020.12.18	1.00	70.00	60.00	1.00	0.00	0.00	0.00	0.00	0.00	3.00	45.00	47.00	1	1.00	#NULL!	416.00	383.00	0.00	0.00	0.00	#NULL!
103.00	249.00	2021.9.1	0.00	78.00	4.00	1.00	0.00	1.00	0.00	0.00	0.00	4.00	45.00	57.00	2C	1.00	#NULL!	352.00	1959.00	0.00	0.00	0.00	#NULL!
104.00	240.00	2021.9.10	0.00	50.00	24.00	0.00	0.00	0.00	0.00	0.00	0.00	0.00	45.00	64.00	1	1.00	#NULL!	#NULL!	974.00	0.00	1.00	1.00	3.00
105.00	960.00	2019.9.21	0.00	66.00	24.00	0.00	0.00	0.00	0.00	0.00	0.00	1.00	44.00	64.00	+box	0.00	0.00	397.00	647.00	0.00	0.00	0.00	#NULL!
106.00	960.00	2019.9.21	0.00	31.00	6.00	0.00	0.00	0.00	0.00	0.00	0.00	0.00	44.00	66.00	1	0.00	0.00	327.00	121.00	0.00	1.00	1.00	6.00
107.00	846.00	2020.1.13	0.00	64.00	1.00	0.00	1.00	0.00	0.00	0.00	0.00	1.00	44.00	62.00	1	0.00	#NULL!	375.00	249.00	0.00	0.00	0.00	#NULL!
108.00	806.00	2020.2.22	0.00	60.00	96.00	0.00	1.00	0.00	0.00	1.00	0.00	3.00	44.00	65.00	1	0.00	#NULL!	477.00	157.00	0.00	0.00	0.00	#NULL!
109.00	247.00	2021.9.3	0.00	71.00	96.00	1.00	1.00	0.00	0.00	0.00	0.00	3.00	44.00	42.00	2C	0.00	#NULL!	598.00	411.00	1.00	0.00	1.00	1.00
110.00	1063.00	2019.6.10	1.00	74.00	6.00	0.00	1.00	1.00	1.00	0.00	1.00	6.00	44.00	45.00	1	1.00	0.00	276.00	3618.00	0.00	0.00	0.00	#NULL!
111.00	1012.00	2019.7.31	0.00	69.00	12.00	0.00	0.00	0.00	1.00	1.00	1.00	5.00	44.00	58.00	1	1.00	0.00	420.00	569.00	0.00	1.00	1.00	12.00
112.00	975.00	2019.9.6	0.00	74.00	120.00	0.00	1.00	0.00	1.00	0.00	1.00	4.00	44.00	64.00	+svc	1.00	0.00	342.00	522.00	0.00	0.00	0.00	#NULL!
113.00	954.00	2019.9.27	0.00	53.00	48.00	0.00	0.00	0.00	0.00	0.00	0.00	0.00	44.00	69.00	1	1.00	0.00	482.00	693.00	0.00	0.00	0.00	#NULL!
114.00	940.00	2019.10.11	0.00	49.00	2.00	0.00	0.00	0.00	0.00	0.00	0.00	0.00	44.00	69.00	1	1.00	0.00	531.00	231.00	0.00	0.00	0.00	#NULL!
115.00	905.00	2019.11.15	0.00	43.00	84.00	0.00	0.00	1.00	0.00	0.00	0.00	1.00	44.00	58.00	1	1.00	#NULL!	444.00	175.00	0.00	0.00	0.00	#NULL!
116.00	902.00	2019.11.18	0.00	63.00	108.00	0.00	1.00	1.00	0.00	0.00	0.00	2.00	44.00	58.00	1	1.00	#NULL!	389.00	134.00	0.00	0.00	0.00	#NULL!
117.00	549.00	2020.11.5	0.00	64.00	3.00	0.00	0.00	0.00	0.00	0.00	0.00	0.00	44.00	68.00	1	1.00	#NULL!	435.00	395.00	0.00	0.00	0.00	#NULL!
118.00	508.00	2020.12.16	0.00	74.00	5.00	0.00	0.00	1.00	0.00	0.00	0.00	2.00	44.00	66.00	1	1.00	#NULL!	459.00	1019.00	0.00	0.00	0.00	#NULL!
119.00	494.00	2020.12.30	0.00	57.00	60.00	0.00	0.00	0.00	0.00	0.00	0.00	0.00	44.00	64.00	1	1.00	#NULL!	473.00	1291.00	0.00	0.00	0.00	#NULL!
120.00	244.00	2021.9.6	1.00	56.00	36.00	0.00	0.00	0.00	0.00	0.00	0.00	1.00	44.00	62.00	1	1.00	#NULL!	347.00	#NULL!	0.00	0.00	0.00	#NULL!
121.00	170.00	2021.11.19	0.00	72.00	6.00	0.00	1.00	0.00	0.00	0.00	0.00	2.00	44.00	67.00	1	1.00	#NULL!	487.00	806.00	1.00	0.00	1.00	1.00
122.00	956.00	2019.9.25	0.00	55.00	120.00	0.00	1.00	1.00	0.00	0.00	0.00	2.00	43.00	64.00	1	0.00	0.00	357.00	628.00	0.00	0.00	0.00	#NULL!
123.00	690.00	2020.6.17	0.00	62.00	48.00	0.00	1.00	0.00	1.00	0.00	1.00	3.00	43.00	53.00	1	0.00	#NULL!	687.50	1032.00	1.00	0.00	1.00	2.00
124.00	643.00	2020.8.3	0.00	68.00	48.00	0.00	0.00	0.00	0.00	0.00	0.00	1.00	43.00	67.00	1	0.00	#NULL!	410.00	420.00	0.00	0.00	0.00	#NULL!
125.00	629.00	2020.8.17	0.00	42.00	12.00	0.00	1.00	0.00	0.00	0.00	1.00	2.00	43.00	73.00	1	0.00	#NULL!	413.00	460.00	0.00	0.00	0.00	#NULL!
126.00	352.00	2021.5.21	1.00	71.00	48.00	0.00	1.00	0.00	0.00	0.00	0.00	3.00	43.00	58.00	+CAFE	0.00	#NULL!	#NULL!	506.00	0.00	0.00	0.00	#NULL!
127.00	1132.00	2019.4.2	0.00	61.00	0.25	1.00	0.00	0.00	0.00	0.00	1.00	2.00	43.00	46.00	2C+Isthmus of TVA	1.00	0.00	#NULL!	1225.00	0.00	0.00	0.00	#NULL!
128.00	1105.00	2019.4.29	1.00	56.00	1.00	0.00	0.00	1.00	0.00	0.00	0.00	2.00	43.00	71.00	1	1.00	0.00	394.30	242.30	0.00	0.00	0.00	#NULL!
129.00	860.00	2019.12.30	1.00	69.00	12.00	0.00	1.00	0.00	1.00	0.00	1.00	5.00	43.00	61.00	1	1.00	#NULL!	474.00	1161.00	0.00	0.00	0.00	#NULL!
130.00	842.00	2020.1.17	0.00	56.00	36.00	0.00	0.00	0.00	0.00	0.00	0.00	0.00	43.00	63.00	1	1.00	#NULL!	434.00	246.00	0.00	0.00	0.00	6.00
131.00	753.00	2020.4.15	0.00	68.00	48.00	0.00	0.00	0.00	0.00	1.00	1.00	4.00	43.00	63.00	1	1.00	#NULL!	465.00	774.00	0.00	1.00	1.00	5.00
132.00	416.00	2021.3.18	1.00	73.00	10.00	0.00	1.00	0.00	0.00	1.00	1.00	6.00	43.00	57.00	+CS	1.00	#NULL!	343.00	1292.00	0.00	0.00	0.00	#NULL!
133.00	361.00	2021.5.12	0.00	66.00	36.00	0.00	0.00	0.00	0.00	0.00	0.00	1.00	43.00	58.00	1	1.00	#NULL!	434.00	3097.00	1.00	0.00	1.00	1.00
134.00	602.00	2020.9.13	0.00	66.00	1.00	0.00	0.00	1.00	0.00	0.00	0.00	2.00	42.30	62.10	1	0.00	#NULL!	238.00	277.00	0.00	0.00	0.00	#NULL!
135.00	1142.00	2019.3.23	1.00	70.00	36.00	1.00	0.00	0.00	0.00	0.00	0.00	3.00	42.00	48.00	1	0.00	0.00	437.00	524.80	0.00	0.00	0.00	#NULL!
136.00	293.00	2021.7.19	0.00	77.00	360.00	0.00	0.00	0.00	1.00	0.00	1.00	4.00	42.00	64.00	1	0.00	#NULL!	425.00	912.00	1.00	0.00	1.00	1.00
137.00	258.00	2021.8.23	1.00	59.00	0.50	0.00	0.00	0.00	0.00	0.00	0.00	1.00	42.00	70.00	1	0.00	#NULL!	#NULL!	#NULL!	0.00	0.00	0.00	#NULL!
138.00	1089.00	2019.5.15	0.00	56.00	2.00	0.00	1.00	0.00	0.00	0.00	0.00	1.00	42.00	65.00	1	1.00	0.00	440.00	403.50	0.00	0.00	0.00	#NULL!
139.00	956.00	2019.9.25	0.00	46.00	0.50	0.00	0.00	0.00	0.00	0.00	0.00	0.00	42.00	60.00	1	1.00	0.00	490.00	136.00	0.00	0.00	0.00	#NULL!
140.00	769.00	2020.3.30	0.00	74.00	1.00	0.00	1.00	1.00	0.00	0.00	1.00	4.00	42.00	62.00	1	1.00	#NULL!	399.00	1325.00	0.00	0.00	0.00	#NULL!
141.00	739.00	2020.4.29	1.00	46.00	36.00	0.00	1.00	0.00	0.00	0.00	0.00	2.00	42.00	46.00	1	1.00	#NULL!	499.00	1386.00	0.00	0.00	0.00	#NULL!
142.00	424.00	2021.3.10	0.00	62.00	2.00	0.00	0.00	0.00	0.00	0.00	0.00	0.00	42.00	60.00	1	1.00	#NULL!	475.00	567.00	0.00	0.00	0.00	#NULL!
143.00	1000.00	2019.8.12	0.00	41.00	240.00	0.00	0.00	1.00	0.00	0.00	0.00	1.00	41.00	64.00	1	0.00	0.00	487.00	634.00	0.00	1.00	1.00	4.00
144.00	725.00	2020.5.13	0.00	72.00	12.00	0.00	0.00	0.00	0.00	0.00	1.00	2.00	41.00	67.00	1	0.00	#NULL!	305.00	945.00	0.00	0.00	0.00	#NULL!
145.00	704.00	2020.6.3	0.00	56.00	2.00	0.00	0.00	0.00	0.00	0.00	0.00	0.00	41.00	71.00	1	0.00	#NULL!	415.00	81.00	0.00	0.00	0.00	#NULL!
146.00	986.00	2019.8.26	0.00	54.00	120.00	0.00	0.00	0.00	0.00	0.00	0.00	0.00	41.00	47.00	1	1.00	0.00	552.00	168.00	0.00	0.00	0.00	#NULL!
147.00	767.00	2020.4.1	0.00	42.00	1.00	0.00	0.00	0.00	0.00	0.00	0.00	0.00	41.00	66.00	1	1.00	#NULL!	422.00	#NULL!	0.00	1.00	1.00	8.00

150.00	354.00	2021.5.19	1.00	43.00	1.00	0.00	0.00	0.00	0.00	0.00	0.00	1.00	41.00	64.00	+CAFE	1.00	#NULL!	307.00	200.00	0.00	0.00	0.00	#NULL!
151.00	310.00	2021.7.2	1.00	73.00	1.00	0.00	0.00	0.00	0.00	0.00	0.00	2.00	41.00	63.00	1	1.00	#NULL!	365.00	619.00	0.00	0.00	0.00	#NULL!
152.00	879.00	2019.12.11	0.00	68.00	18.00	0.00	1.00	0.00	0.00	0.00	0.00	2.00	40.00	74.00	+svc	0.00	#NULL!	476.00	131.00	0.00	1.00	1.00	12.00
153.00	1106.00	2019.4.28	0.00	35.00	0.50	0.00	0.00	0.00	0.00	0.00	0.00	0.00	40.00	57.00	1	1.00	0.00	298.00	176.70	1.00	1.00	1.00	3.00
154.00	776.00	2020.3.23	1.00	73.00	3.00	0.00	0.00	1.00	0.00	0.00	0.00	3.00	40.00	66.00	1	1.00	#NULL!	374.00	2523.00	0.00	0.00	0.00	#NULL!
155.00	419.00	2021.3.15	1.00	50.00	0.50	1.00	0.00	0.00	0.00	0.00	1.00	3.00	40.00	52.00	1	1.00	#NULL!	567.00	1464.00	0.00	0.00	0.00	#NULL!
156.00	347.00	2021.5.26	0.00	61.00	6.00	0.00	0.00	0.00	0.00	0.00	1.00	1.00	40.00	69.00	1	1.00	#NULL!	413.00	398.00	0.00	0.00	0.00	#NULL!
157.00	335.00	2021.6.7	0.00	65.00	24.00	0.00	0.00	0.00	0.00	0.00	0.00	1.00	40.00	57.00	1	1.00	#NULL!	425.00	624.00	0.00	0.00	0.00	#NULL!
158.00	935.00	2019.10.16	0.00	58.00	60.00	0.00	1.00	0.00	0.00	0.00	0.00	1.00	39.00	39.00	1	0.00	0.00	415.00	292.00	0.00	0.00	0.00	#NULL!
159.00	284.00	2021.7.28	0.00	40.00	36.00	0.00	0.00	0.00	0.00	0.00	0.00	0.00	39.00	61.00	1	0.00	#NULL!	301.00	#NULL!	1.00	0.00	1.00	1.00
160.00	1104.00	2019.4.30	0.00	68.00	6.00	0.00	0.00	1.00	0.00	0.00	0.00	2.00	39.00	61.00	1	1.00	0.00	382.30	408.60	0.00	0.00	0.00	#NULL!
161.00	979.00	2019.9.2	0.00	63.00	36.00	0.00	0.00	1.00	0.00	0.00	0.00	1.00	39.00	60.00	1	1.00	0.00	326.00	287.00	0.00	0.00	0.00	#NULL!
162.00	958.00	2019.9.23	0.00	38.00	0.50	0.00	0.00	0.00	0.00	0.00	0.00	0.00	39.00	59.00	1	1.00	0.00	644.00	681.00	0.00	1.00	1.00	9.00
163.00	303.00	2021.7.9	1.00	61.00	1.00	0.00	1.00	0.00	0.00	0.00	0.00	2.00	39.00	66.00	1	1.00	#NULL!	280.00	719.00	1.00	0.00	1.00	1.00
164.00	1042.00	2019.7.1	1.00	37.00	24.00	0.00	0.00	0.00	0.00	0.00	0.00	1.00	38.00	57.00	1	0.00	0.00	336.00	306.00	0.00	0.00	0.00	#NULL!
165.00	179.00	2021.11.10	0.00	74.00	240.00	1.00	0.00	0.00	0.00	1.00	1.00	5.00	38.00	76.00	1	0.00	#NULL!	345.00	513.00	0.00	0.00	0.00	#NULL!
166.00	1005.00	2019.8.7	1.00	38.00	96.00	0.00	0.00	0.00	0.00	0.00	0.00	1.00	38.00	59.00	1	1.00	0.00	659.00	664.00	0.00	1.00	1.00	8.00
167.00	893.00	2019.11.27	0.00	53.00	0.50	0.00	0.00	1.00	0.00	0.00	0.00	1.00	38.00	65.00	1	1.00	#NULL!	377.00	222.00	0.00	0.00	0.00	#NULL!
168.00	865.00	2019.12.25	0.00	62.00	60.00	0.00	0.00	0.00	0.00	0.00	0.00	0.00	38.00	69.00	1	1.00	#NULL!	498.00	492.00	0.00	1.00	1.00	30.00
169.00	849.00	2020.1.10	0.00	50.00	48.00	0.00	0.00	0.00	0.00	0.00	0.00	0.00	38.00	75.00	1	1.00	#NULL!	327.00	435.00	0.00	0.00	0.00	#NULL!
170.00	468.00	2021.1.25	0.00	56.00	2.00	0.00	0.00	0.00	0.00	0.00	0.00	0.00	38.00	49.00	1	1.00	#NULL!	300.00	159.00	0.00	0.00	0.00	#NULL!
171.00	403.00	2021.3.31	0.00	52.00	4.00	0.00	0.00	0.00	0.00	0.00	0.00	0.00	38.00	60.00	1	1.00	#NULL!	389.00	297.00	0.00	0.00	0.00	#NULL!
172.00	419.00	2021.3.15	0.00	50.00	1.00	0.00	0.00	1.00	0.00	0.00	0.00	1.00	37.00	62.00	1	1.00	#NULL!	316.00	#NULL!	0.00	0.00	0.00	#NULL!
173.00	408.00	2021.3.26	0.00	67.00	1.00	0.00	0.00	0.00	0.00	0.00	1.00	2.00	37.00	64.00	1	1.00	#NULL!	436.00	518.00	1.00	0.00	1.00	1.00
174.00	671.00	2020.7.6	0.00	36.00	2.00	0.00	0.00	0.00	0.00	0.00	0.00	0.00	36.00	71.00	1	0.00	#NULL!	563.00	280.00	0.00	0.00	0.00	#NULL!
175.00	1035.00	2019.7.8	1.00	64.00	12.00	0.00	0.00	0.00	0.00	0.00	0.00	1.00	36.00	66.00	1	1.00	0.00	208.00	274.00	0.00	0.00	0.00	#NULL!
176.00	844.00	2020.1.15	0.00	78.00	0.30	0.00	0.00	0.00	0.00	0.00	0.00	2.00	36.00	58.00	1	1.00	#NULL!	364.00	1340.00	0.00	1.00	1.00	5.00
177.00	746.00	2020.4.22	0.00	66.00	72.00	0.00	0.00	0.00	0.00	0.00	0.00	1.00	36.00	44.00	1	1.00	#NULL!	422.00	1101.00	0.00	0.00	0.00	#NULL!
178.00	725.00	2020.5.13	0.00	62.00	0.50	0.00	1.00	0.00	0.00	0.00	0.00	1.00	36.00	63.00	+epicardium	1.00	#NULL!	#NULL!	257.00	0.00	1.00	1.00	8.00
179.00	170.00	2021.11.19	0.00	79.00	12.00	0.00	1.00	0.00	0.00	0.00	0.00	3.00	36.00	63.00	2C	1.00	#NULL!	646.00	380.00	0.00	0.00	0.00	#NULL!
180.00	1082.00	2019.5.22	0.00	54.00	24.00	0.00	0.00	0.00	0.00	0.00	0.00	0.00	35.00	65.00	1	1.00	0.00	532.00	933.00	0.00	0.00	0.00	#NULL!
181.00	695.00	2020.6.12	1.00	37.00	12.00	0.00	0.00	0.00	0.00	0.00	0.00	1.00	35.00	65.00	1	1.00	#NULL!	328.00	309.00	0.00	0.00	0.00	#NULL!
182.00	475.00	2021.1.18	0.00	50.00	24.00	0.00	0.00	0.00	0.00	0.00	0.00	0.00	35.00	59.10	+CAFE	1.00	#NULL!	297.00	363.00	0.00	0.00	0.00	#NULL!
183.00	200.00	2021.10.20	1.00	57.00	1.00	0.00	1.00	0.00	0.00	0.00	0.00	2.00	35.00	62.00	2C	1.00	#NULL!	#NULL!	1067.00	0.00	0.00	0.00	#NULL!
184.00	128.00	2021.12.31	1.00	50.00	480.00	0.00	0.00	0.00	0.00	0.00	0.00	1.00	35.00	63.00	2C	1.00	#NULL!	438.00	229.00	1.00	0.00	1.00	1.00
185.00	660.00	2020.7.17	1.00	31.00	84.00	0.00	0.00	0.00	0.00	0.00	0.00	1.00	34.00	62.00	+svc	0.00	#NULL!	397.00	354.00	0.00	0.00	0.00	#NULL!
186.00	942.00	2019.10.9	0.00	48.00	1.00	0.00	0.00	0.00	0.00	0.00	0.00	0.00	34.00	63.00	+epicardium	1.00	0.00	394.00	273.00	0.00	0.00	0.00	#NULL!
187.00	163.00	2021.11.26	1.00	75.00	6.00	0.00	0.00	0.00	0.00	0.00	1.00	4.00	34.00	67.00	2C	1.00	#NULL!	422.00	1279.00	0.00	0.00	0.00	#NULL!
188.00	312.00	2021.6.30	0.00	69.00	0.33	0.00	0.00	0.00	0.00	0.00	0.00	1.00	31.00	74.00	1	1.00	#NULL!	454.00	835.00	0.00	0.00	0.00	#NULL!
189.00	354.00	2021.5.19	1.00	63.00	2.00	0.00	0.00	0.00	0.00	0.00	0.00	1.00	#NULL!	#NULL!	1	0.00	#NULL!	346.00	421.00	0.00	1.00	1.00	6.00
190.00	324.00	2021.6.18	1.00	70.00	240.00	0.00	0.00	0.00	0.00	0.00	0.00	2.00	#NULL!	69.00	1	0.00	#NULL!	384.00	888.00	1.00	0.00	1.00	1.00
191.00	422.00	2021.3.12	0.00	66.00	120.00	0.00	1.00	0.00	1.00	0.00	0.00	3.00	#NULL!	#NULL!	1	0.00	#NULL!	247.00	588.00	0.00	0.00	0.00	#NULL!
192.00	863.00	2019.12.27	0.00	74.00	12.00	0.00	1.00	0.00	0.00	0.00	0.00	2.00	#NULL!	#NULL!	1	1.00	#NULL!	531.00	934.00	0.00	0.00	0.00	#NULL!
